# A Method to Study the Epigenetic Chromatin States of Rare Hematopoietic Stem and Progenitor Cells; MiniChIP–Chip

**DOI:** 10.1007/s12575-010-9031-y

**Published:** 2010-05-15

**Authors:** Holger Weishaupt, Joanne L Attema

**Affiliations:** 1 Immunology Unit, Institute for Experimental Medical Science, BMC D14, Lund University, 221 84, Lund, Sweden

**Keywords:** Miniaturized chromatin immunoprecipitation assays, Microarray technology, Histone modifications, Stem and progenitor cells, Epigenetic regulation, Lineage commitment

## Abstract

Dynamic chromatin structure is a fundamental property of gene transcriptional regulation, and has emerged as a critical modulator of physiological processes during cellular differentiation and development. Analysis of chromatin structure using molecular biology and biochemical assays in rare somatic stem and progenitor cells is key for understanding these processes but poses a great challenge because of their reliance on millions of cells. Through the development of a miniaturized genome-scale chromatin immunoprecipitation method (miniChIP–chip), we have documented the genome-wide chromatin states of low abundant populations that comprise hematopoietic stem cells and immediate progeny residing in murine bone marrow. In this report, we describe the miniChIP methodology that can be used for increasing an understanding of the epigenetic mechanisms underlying hematopoietic stem and progenitor cell function. Application of this method will reveal the contribution of dynamic chromatin structure in regulating the function of other somatic stem cell populations, and how this process becomes perturbed in pathological conditions.

## 1 Introduction

A unique and defining property for any stem cell population is intrinsic self-renewal throughout the process of cell division, whilst maintaining the capacity to form multiple differentiated and mature cell types over the lifetime of an organism. Stem cells undergo dramatic changes in morphology, cell cycle status and gene expression during differentiation into specialized progenitor subsets. Such alterations are proposed to result from chromatin reorganization of the genome, allowing for the establishment and maintenance of lineage-specific transcriptional networks [1,2]. Therefore, cellular differentiation can be viewed as a product of heritable chromatin states, which in turn, are induced and maintained by specific epigenetic chromatin modifications [3].

Chromatin structure defines the higher order structure by which DNA is organized within the cell nucleus [4]. It consists of a chain of nucleosomes, representing about 146–147 bp of DNA wrapped around a core of histone octamers [5]. The precise degree of nucleosome compaction at genomic regions influences the accessibility of transcription factor binding to gene promoters and enhancer regions critical for transcriptional regulation [6]. Accordingly, genomic regions are packaged into euchromatin, which forms a relaxed structure of large genomic distances between nucleosomes, or a higher degree of DNA compaction known as heterochromatin [7]. Euchromatin is also referred to as active chromatin and is linked to actively transcribed regions of the genome, while genes located in heterochromatin (inactive chromatin) are usually transcriptionally silent.

In mammals, the most studied epigenetic chromatin modifications are DNA methylation and covalent modifications at the N-terminal tails of histone proteins [8,9]. DNA cytosine methylation, as mediated by the DNA methyltransferases, is thus far the only epigenetic mechanism known to directly modify DNA [10,11]. DNA methylation occurs mainly on cytosine–phosphate–guanine dinucleotides that are associated with transcriptionally inactive heterochromatic regions to mediate stable gene silencing. Histone proteins including their N-terminal tails regions are subject to a variety of different post translational modifications, including methylation, acetylation, phosphorylation, ubiquitylation, sumoylation, ADP ribosylation, glycosylation, biotinylation, and carbonylation [12]. Unlike DNA methylation, which has been linked gene silencing, these diverse histone modifications show differential patterns of activating and silencing effects on gene transcription. For example, histone acetylation is almost entirely associated with gene activation, while lysine methylation can lead to both gene activation and silencing depending on the modified lysine residue and the degree of methylation (mono-, di-, or trimethylation). To date, over 100 possible histone modifications have been identified. Accumulating evidence reveals that most histones are simultaneously modified by a combination of different modifications, and directly supports the histone code hypothesis [13-15]. Recent efforts in deciphering this highly complex code in cellular differentiation are beginning to shed light on the molecular mechanisms of lineage commitment and differentiation potential during development [16-20].

A gold standard approach for the investigation of epigenetic chromatin states is the chromatin immunoprecipitation (ChIP) assay. ChIP is a powerful method that directly permits the measurement of in vivo DNA–protein interactions, through the use of antibodies targeted against specific DNA-associated proteins and thus enriching for the bound DNA fragments [21,22]. Recently, ChIP has been combined with microarray (ChIP–chip) and high throughput sequencing (ChIP–Seq) technologies to perform genome-wide analysis of transcription factor and histone modification association at genomic regions in a wide range of cell types including embryonic and adult stem cell populations [8,20,23-25]. However, traditional ChIP protocols require a large amount of cells for starting material (typically >1 × 10^7^ cells), which has frequently limited such analyses to primary cells differentiated in culture, cell lines, whole tissues, and primary cells from in vivo sources.

To overcome this technical hindrance, we and others have recently developed modified ChIP assays allowing for loci-specific and global-based analyses to be performed with cell numbers ranging from 100,000 to as few as 1,000 cells [26-28]. These technical developments have proven to be critical for positioning this method into the cell number range of primary stem and progenitor cell populations that normally occur in vivo. Typically, primary stem cell populations responsible for maintaining tissues and organs occur at a very low frequency. Our method that we refer to as miniChIP–chip has enabled for the analysis of 10,000 primary highly purified hematopoietic stem cell populations, which are present in the bone marrow at a frequency of 0.0001% [28]. Here we describe the detailed methods to investigate the chromatin states of murine hematopoietic stem cells (HSCs), early progenitors, and the bioinformatics approaches used to identify novel regulators of hematopoiesis with miniChIP–chip and gene expression microarrays. The miniChIP–chip technology described in this method article could be applied in studies that aim to investigate the chromatin states of other somatic stem cell compartments during normal physiology, rare primary cell populations obtained from pathological tissues, as well as cell suspensions obtained from model organisms such as zebrafish, fruitfly, and nematodes.

## 2 Materials and Methods

### 2.1 Reagents and Equipment

Hanks buffered salt solution (HBSS 14175-053), Dulbecco's modified eagle medium (DMEM #31966-021), Dulbecco's phosphate-buffered saline (DPBS 14190-094) were purchased from Gibco. Fetal calf serum (FCS) was purchased from Hyclone Laboratories. CD117 (ckit) microbeads (130-091-224) was purchased from Miltenyi Biotech. Dynabeads^®^ Protein A (100.02D), phenol/chloroform/isoamyl alcohol (15593-049) and SYBR^®^ GreenER mastermix (11761-500), Superscript III First strand synthesis system (18080-051) and 1 mg/mL Propidium Iodide (Molecular Probes, P3566) were purchased from Invitrogen. Complete protease inhibitor cocktail (PIC, 11697498001) and Proteinase K (03115828001) were purchased from Roche. Thirty-seven percent formaldehyde solution (F8775), β-mercaptoethanol (63689), and GenomePlex^®^ whole genome amplification kits (WGA2 and WGA4) were purchased from Sigma Aldrich. Linear acylamide (AM9520), glycogen (AM9510), and DNaseI (AM2222) were purchased from Ambion. ChIP antibodies were purchased from the following companies, anti-H3K4me3 (Upstate MC315 and Abcam ab8580); anti-H3K79me2 (Upstate NL59); anti-H3K27me3 (Abcam 07-499); anti-H3K9me3 (Abcam ab8898); and anti-PolII (Active Motif 39097). Sterile barrier tips with low retention surface were purchased from CLP Neptune (BT1000, BT200, BT20, and BT10XL). Eppendorf tubes (1.5 ml, maximum recovery MCT-150-LC) were purchased from Axygen, whereas 0.2 ml PCR tubes were purchased from Sarstedt (72.737.002). A sonication water bath, Bioruptor^®^ (UCD-200), was purchased from Diagenode.

### 2.2 Isolation of Primary Hematopoietic Cells

Murine bone marrow was harvested from the two femurs, tibias, and hips of 10–12-week-old C57Bl/6 mice following consent from the Lund University ethics committee. Bone marrow cells (BMC) were harvested by crushing bones into 10 ml of DPBS containing 2% FCS (DPBS/FCS). Harvested BMCs were filtered through 70 μM cell strainers (BD falcon) into 50 ml tubes. Bone fragments were rinsed with an additional 10 ml of DPBS/FCS, pooled into 50 ml tubes, and centrifuged at 1,200 rpm (300 rcf) for 10 min at 4°C. The supernatant was discarded and pellets were gently resuspended in 10 ml of DPBS/FCS. The cell suspension was filtered through new 70 μM cell strainers into new 50 ml tubes, and centrifuged at 1,200 rpm (300 rcf) for 10 min at 4°C. The supernatant was removed and the resultant BMC pellet was gently resuspended in 100 μl of DPBS/FCS per mouse ready for c-kit enrichment. HSCs, MPPs, and PreMegEs were positively selected using c-kit conjugated magnetic beads (Miltenyi, Germany) according to manufacturer's procedures except that 3.5 μl of the bead slurry per mouse was used, and ckit-bead incubation was performed on ice for 30 min. Following positive selection, cells were stained in 0.5 ml of DPBS/FCS with directly conjugated antibodies in the dark for 30 min on ice. The following antibodies were used; anti-Sca-1-Pacific Blue (D7, Biolegend), anti-c-kit-APC/AL780 (2B8, eBioscience), anti-Slamf1-APC (CD150; TC15-12F12.2, Biolegend), anti-CD41-FITC (MWReg30; Becton Dickinson), anti-CD105-PeCy7 (MJ7/18 eBioscience), anti-Flt3-PE (A2F10, eBioscience), and the lineage antibodies against anti-CD4 (GK1.5), anti-CD8 (53–6.7), anti-B220 (RA3-6B2), anti-Gr1 (RB6-8C5), anti-Mac1 (M1/70), anti-Ter119 (Ter119) directly conjugated to PECy5 or indirectly to QD605 using biotin/streptavidin conjugation. Anti-CD48-FITC (HM48-1 Biolegend) antibodies were used in place of anti-CD41-FITC when performing HSC and MPP analysis as previously described [29]. Propidium iodide was used to discriminate dead cells (1 μg/ml in DPBS/FCS). Cells were maintained on ice when possible through all procedures and were sorted into maximum recovery 1.5 ml Eppendorf tubes (Axygen) using the single sort mode on a FACS Aria cell sorter (Becton Dickinson). Post-sorting reanalysis routinely showed >95% purity. All flow cytometry and FACS data were analyzed with FlowJo software (Treestar, Ashland, OR).

### 2.3 10,000 Cell MiniChIP Assay

The 10,000 cell miniaturized chromatin immunoprecipitation qPCR (miniChIP–qPCR) method was established from previously described protocols [30,31]. It was critical that each 100 μl lysate represented 10,000 cells otherwise the assay showed variation between replicate experiments. Low retention barrier tips and Eppendorf tubes were used in all steps. Careful pipetting of solutions was employed throughout, and the pipetting of solutions up and down into reactions was avoided. The procedure was conducted in a time efficient manner without long breaks between the steps. The concentrated stocks of buffers (e.g., 1 M Tris, 0.5 M EDTA, 5 M NaCl, 10% sodium dodecyl sulfate (SDS)) were autoclaved and stored at room temperature. The ChIP buffers were conveniently made in sterile 50 ml Falcon tubes and were 0.2 μM filter sterilized and subsequently stored at 4°C. All steps were performed on ice or in a cold room unless otherwise stated. The following procedure outlines a method for processing one ChIP reaction comprising 10,000 cells. This procedure was scaled up to 50,000, 80,000, or 100,000 cells at the formaldehyde crosslinking step depending on the experimental design (see Step-wise Protocol for a miniChIP method that describes an experiment starting with 30,000 cells).

**Figure 2:**
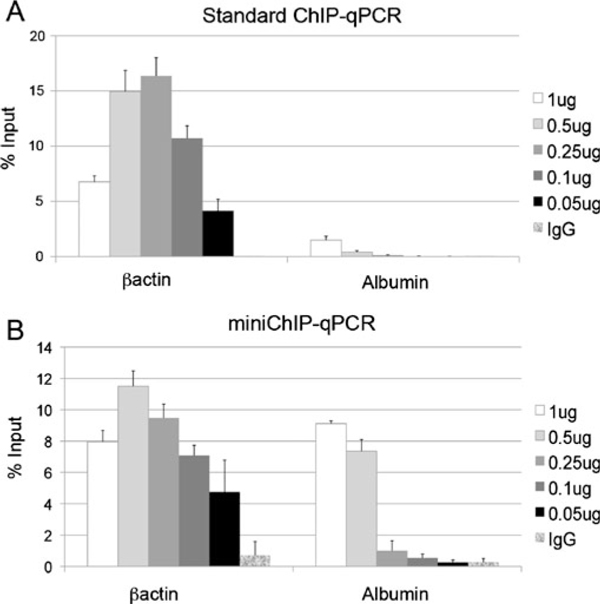
**Titration analysis of an anti-H3K4me3 antibody in standard and scaled miniChIP assays**. Assessment of the anti-H3K4me3 antibody (Upstate 04-745) in standard ChIP (**a**) and miniChIP (**b**) assays using the βactin and albumin promoters across a titration range of 1–0.05 μg. Standard and miniChIP assays were optimized for the analysis of 1 × 10^6^ and 10,000 cells per reaction, respectively. H3K4me3 enrichment is represented as % input at gene promoters (*y*-axis) obtained from the murine embryonic fibroblasts. % input was determined as described in "Materials and Methods", and was used for the analysis of ChIP–qPCR data in all subsequent figures. Low background signals were detected with the IgG control sera at both βactin and albumin promoters. *Error bars* represent the standard deviation (±SD) of at least three independent ChIP assays.

Briefly, 10,000 primary cells were crossed-linked in a final concentration of 0.1% formaldehyde in 1 ml of DMEM containing 10% FCS at room temperature for 10 min and washed once with ice-cold HBSS containing protease inhibitor cocktail (HBSS/PIC) and stored at -80°C. Ten thousand cross-linked cells were thawed on ice and lysed in 25 μl Lysis buffer (10 mM Tris pH 7.5/1 mM EDTA/1% SDS) for 5 min, then diluted with 75 μl of HBSS/PIC and the 100 μl volume was sonicated for five cycles of 30 s with a 30 s rest period in between cycles (Bioruptor^®^ Diagenode). PIC was used in all buffers throughout the procedure. The sonicated chromatin was microcentrifuged for 5 min at 13,000 rpm (17,000 rcf) at 4°C to remove precipitated SDS and 120 μl of 2× RIPA buffer (20 mM Tris pH 7.5/2 mM EDTA/2%Triton X-100/0.1% SDS/0.2% sodium deoxycholate/200 mM NaCl) was added to recovered supernatants. A 1/10 volume (20 μl) was removed for input control. ChIP-qualified antibodies were added to the sonicated chromatin and incubated at 4°C for 2 h. The amounts of antibodies were empirically determined in antibody titration experiments (Figures 2, S1, S2). This step was carried out up to 16 h depending on the efficiency of the antibody. However, prolonged incubation times increased non-specific antibody binding and chromatin degradation, and were generally avoided.

Following antibody incubation, 10 μl of protein A or G Dynabeads^®^ magnetic beads (Dynal, Invitrogen) previously washed in 1× RIPA buffer were added and incubated for an additional 2 h at 4°C. The bead–protein complexes were washed three times with 100 μl of 1× RIPA buffer (10 mM Tris pH 7.5/1 mM EDTA/1%Triton X-100/0.1% SDS/0.1% sodiumdeoxycholate/100 mM NaCl) and once with 100 μl of TE (10 mM Tris pH 7.5/10 mM EDTA) buffer using a magnetic rack to collect the beads. The genomic DNA was then eluted for 2 h at 65°C in 300 μl of Elution Buffer (20 mM Tris pH 7.5/5 mM EDTA/50 mM NaCl/1% SDS/50 μg/ml proteinase K) using a shaking Eppendorf Thermomixer (1,300 rpm). Genomic DNA was recovered into new tubes and purified using phenol/chloroform extraction (300 μl of phenol/chloroform/isoamyl alcohol) and subsequent ethanol precipitation (800 μl of 97% ethanol) using linear acrylamide and glycogen carriers (10 μg of each). Following centrifugation (13,000 rpm (17,000 rcf) for 20 min at 4°C) and 70% ethanol rinse with 400 μl (13,000 rpm (17,000 rcf) for 10 min at 4°C), genomic DNA pellets were air dried and resuspended in 24 μl T_10_E_0.1_ (10 mM Tris pH 7.5/0.1 mM EDTA) buffer, and 1 μl was used in each SYBR green qPCR reaction with gene-specific primers. This enabled 24 individual qPCR reactions or eight genomic regions analyzed in triplicate PCR reactions. Real-time PCR reactions were performed in a BioRad MyiQ sequence detection system using the 2× SYBR green master mix according to manufacturer's instructions (Invitrogen). Enrichment of histone modifications and PolII at genomic regions were expressed as % input using the formula; % (ChIP/total input) = 2^[(*C*_t_(ChIP) - *C*_t_(input) × DF)] × 100%. This calculation was determined using equally efficient SYBR green qPCR primer sets as previously described [28].

### 2.4 10,000 Cell MiniChIP–Chip Technology

The ChIP DNA was prepared as described in the miniChIP–qPCR protocol above except that the DNA pellets were dissolved in 10 μl of T_10_E_0.1_ (10 mM Tris pH 7.5/0.1 mM EDTA) buffer. An entire ChIP sample was subjected to whole genome amplification (WGA) using the WGA4 kit (Sigma Aldrich) using 0.2 ml PCR tubes. Note that the cell lysis and DNA fragmentation steps were omitted and the protocol started at Step 6 of the WGA4 procedure. A matching number of input samples were amplified to cover the number of amplified ChIP DNA samples for array analysis. WGA lead to the production of ~10 μg of amplified ChIP and input DNA samples. Prior to array analysis, sample integrity was confirmed using NanoDrop spectrophotometer and qPCR surveys of positive and negative control genomic regions was conducted. The NimbleGen HD2 Hx.1 delux promoter tiling arrays of ~22,000 RefSeq gene promoters represented in the mouse genome were used for array analysis of amplified miniChIP DNA. Microarray hybridization was performed by NimbleGen Service Facility (Iceland) according to the manufacturer's procedures. Raw and processed data for the miniChIP–chip experiments can be found under accession number GSE18737 super series in the NCBI Gene Expression Omnibus. Bioinformatics scripts used for the data analysis can be obtained by contacting the authors.

### 2.5 Quantitative RT-PCR Analysis

For quantitative RT-PCR (qRT-PCR), approximately 5,000–10,000 purified primary hematopoietic cells were sorted directly into 350 μl of RLT buffer containing 3.5 μl of β-mercaptoethanol (RNeasy micro kit, Qiagen). Samples were maintained on ice during the sorting process, mixed by manual agitation, and immediately snap frozen on dry ice and stored at -80°C until further processing. Total RNA was isolated using an RNeasy micro kit (Qiagen) from purified primary hematopoietic cells using on-column DNaseI treatment according to the manufacturer's instructions. The resultant volume of RNA samples in "EB buffer" following column elution was 10 μl. The samples were additionally digested with 1 μl DNaseI and 1 μl of 10× DNase1 reaction buffer (Ambion) for 20 min at 37°C to remove genomic DNA. DNaseI was inactivated by incubating samples at 65°C for 10 min. The sample (12 μl) was then used for reverse-transcription using random hexamers according to the manufacturer's instructions (SuperScript III RT-PCR system, Invitrogen). The qRT-PCR reactions were performed with 500 cell equivalents of RNA and gene-specific primers in a BioRad MyiQ sequence detection system using the 2× SYBR green master mix according to manufacturer's instructions (Invitrogen).

### 2.6 Affymetrix Gene Expression Experiments

For Affymetrix gene expression microarray experiments, approximately 5,000–10,000 purified primary hematopoietic cells were sorted directly into 350 μl of RLT buffer containing 3.5 μl of β-mercaptoethanol (RNeasy micro kit, Qiagen). Samples were maintained on ice during the sorting process, mixed by manual agitation, and immediately snap frozen on dry ice and stored at -80°C until further processing. RNA was extracted from purified primary hematopoietic cells using the RNeasy micro kit (Qiagen) using on-column DNaseI treatment according to the manufacturer's instructions. The resultant volume of RNA samples in "EB buffer" following column elution was 10 μl. Following quality control assessment using Bioanalyser analysis (Agilent), approximately 10 ng of each RNA sample was subjected to rounds of RNA amplification using the Affymetrix sample labeling kit according to the manufacturer's instructions. Samples were processed (labeling, hybridization, washing, scanning) using the Affymetrix Mus musculus 430 2.0 arrays. Array processing was performed in the SciBlue service Facility (Lund University, Sweden). Raw and processed data for the Affymetrix microarray experiments can be found under accession number GSE18737 super series in the NCBI Gene Expression Omnibus.

## 3 Results and Discussion

### 3.1 MiniChIP–qPCR Assays Based on 10,000 Cells

We have established a streamlined miniChIP–qPCR method that enables for the reproducible analysis of 10,000 cells [28,30,32]. As we reported recently, this was a refinement of our previously described miniChIP–qPCR method, which allowed histone modifications to be identified at selected genes using 50,000 cells [30]. Thus, we made further modifications to enable rapid genome-scale analysis using 10,000 cells. Further reducing the cell number requirements of the miniChIP method was critical in order to study the unusually small numbers of hematopoietic stem cells present in adult murine bone marrow (~2,000 cells/two femurs, tibias, and hip bones per mouse). During the development of this method, two independent reports demonstrated μChIP and microChIP using 1,000 and 10,000 cells, respectively, thus providing additional support for scaled ChIP–chip methods [26,27,33]. In this following section, we discuss the types of reagents and quality controls required for effective implementation of miniChIP–qPCR assays into the research laboratory.

**Figure 1:**
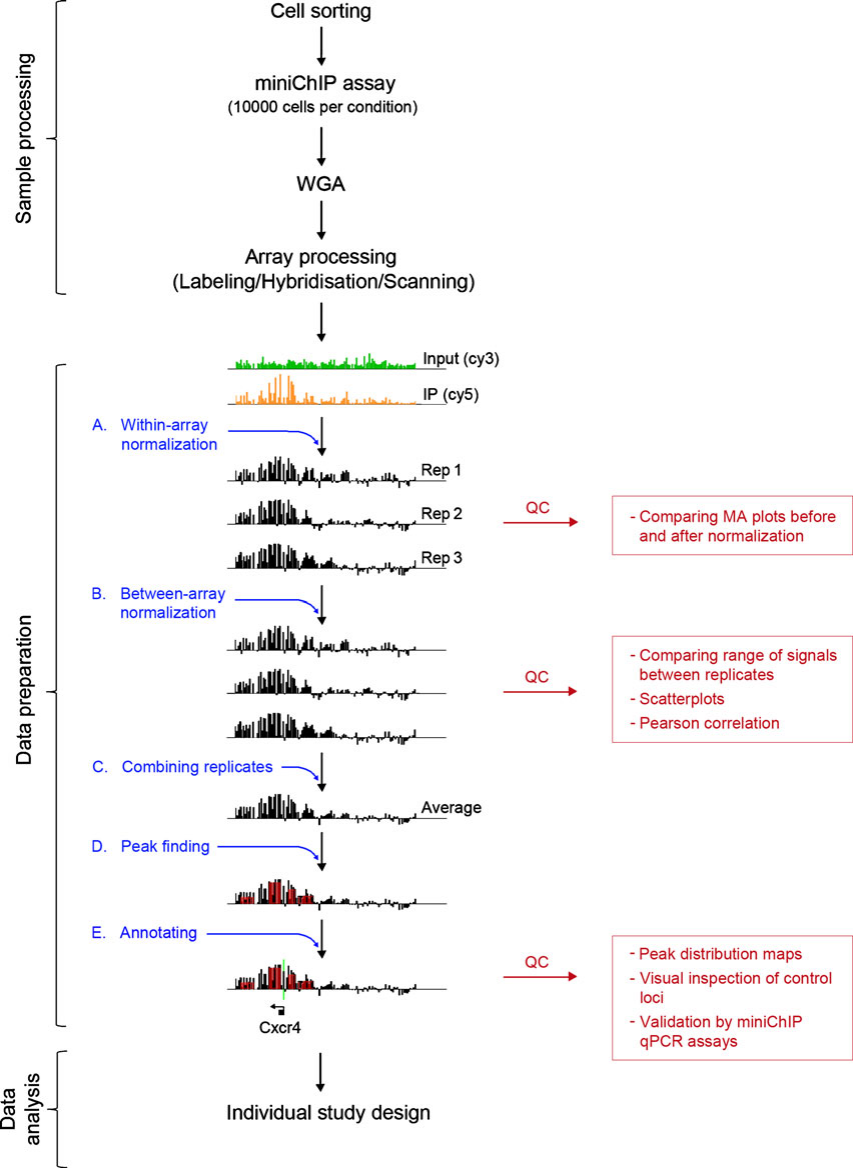
**Technical pipeline for the miniChIP–chip technology platform using 10,000 cells**. The flowchart depicts the three main methodical phases of the miniChIP–chip procedure: sample processing, data preparation, and data analysis. Detailed steps of the data preparation phase (*A*–*E*) is indicated including a visual depiction of the array data and possible quality controls (*QC*). The representative data in *A*–*E* was obtained from miniChIP–chip investigations of H3K4me3 associated with the Cxcr4 locus in murine hematopoietic stem cells (phenotypically defined by fluorescence-activated cell sorting as lineage^-/lo^, ckit^+^, Sca1^+^, CD150^+^, Flk2/Flt3^- ^[28].

In order to scale down our recently described miniChIP–qPCR method from 50,000 cells to 10,000 cells, new reagents and simplified steps were introduced. These refinements lead to a miniChIP–qPCR method that was short, easy, and reliable for use with 10,000 cells per experimental condition (1 day from cells to qPCR analysis of miniChIP DNA; Figure 1; Materials and Methods) [32]. The new assay components included low retention tubes and tips (Axygen), protein A/G magnetic beads (Dynal), water bath sonication, one-step cell lysis, reduced reaction volumes, dilutions, incubation times, and immune complex wash steps. The concentration of formaldehyde used for protein–DNA crosslinking was reduced tenfold compared to other methods that typically employ 1% formaldehyde [26,33]. We found that protein–DNA crosslinking with 0.1% formaldehyde reduced the false positive signals at genomic regions, increased genomic DNA recovery following reversal of crosslinks/DNA purification steps and improved signal to noise ratio (data not shown). The use of a single-step cell lysis approach was critical for further minimizing sample loss. This was in contrast to other scaled methods, which comprised at least two lysis steps for cellular extraction and subsequent lysis [26,33] thereby increasing sample loss. Our method relied on a single incubation of cell pellets with lysis buffer, followed by one tube change in preparation for chromatin shearing using a water bath sonicator. This simplified lysis step reduced sample handling and loss, which would impact on ChIP efficiencies.

The sonication step was a critical component of our ChIP method, and cycle conditions were optimized for each cell quantity used. Sonication analysis using 10,000 cells presented a challenge due to the low amount of DNA recovered for subsequent analysis using agarose gels. Therefore, we processed at least three replicate 10,000 cell reactions for each sonication cycle number tested, and pooled the reactions prior to the DNA recovery steps (Figure S1). This enabled visualization of the sheared DNA on agarose gels as previously described [26]. The use of a sonication bath (Diagenode) was used when dealing with small numbers of cells. This device supported sonication of small reaction volumes by eliminating foaming. In our method, the sonication of 10,000 cells was performed in 100 μl, which comprised 25 μl of lysis buffer and 75 μl of Hanks Buffered Salt Solution (HBBS). Dilution of the cell lysate with HBBS reduced the sodium dodecyl sulfate concentration to 0.25%, which allowed for optimal sonication and immunoprecipitation reaction conditions. Importantly, a twofold dilution with 2XRIPA buffer could be performed instead of tenfold dilution that has been described in the conventional Upstate/Millipore Protocol (cat no. 17-295) as well as other scaled methods [26,33]. Thus, we could perform a highly efficient immunoprecipitation reaction in 200 μl and in other words, tenfold less volume compared to other conventional and scaled methods [34].

The success of miniChIP assays critically depended on the quality assessment of antibodies used during the chromatin immunoprecipitation step. Figure 2 shows a comparison of anti-H3K4me3 antibody in standard ChIP–qPCR (1 × 10^6^ cells per reaction) and miniChIP–qPCR (10,000 cells per reaction) assays. Using standard and miniChIP, the highest enrichment value obtained for anti-H3K4me3 was observed with 0.25 and 0.5 μg, respectively, at the transcriptionally active βactin promoter but not the silent albumin promoter (Figure 2a). However, the optimal amounts of antibody lead to a high degree of non-specific false positive enrichment at the albumin promoter in miniChIP but not the standard assay (Figure 2b). Titration analysis of anti-H3K4me3 revealed that an optimal signal to noise ratio was achieved in miniChIP using twofold less antibody. False positive enrichment at numerous genomic regions occurred when using higher concentrations of ChIP-qualified antibodies specifically in miniChIP assays (data not shown). This probably related to the antibody type and the purification method used since anti-H3K4me3 and anti-H3K27me3 showed this behavior but not anti-H3K36me3 and anti-H3K9me3. The possible cause remains unclear, but could most likely result from using significantly less chromatin in miniChIP reactions compared to the standard assay. Nonetheless, this result emphasized the importance of performing broad titrations of antibodies in order to assess their performance in the modified miniChIP assays compared to the standard protocol.

Careful screening of antibodies from different companies increased the likelihood of finding an antibody suitable for use in the miniChIP assays. For example, anti-H3K27me3 was sourced from Active Motif (AM174), Millipore (07-499), and Abcam (ab6002). As shown previously and in Figure S2, H3K27me3 associated with the p16^Ink4a^ and Myt-1 loci in early passage murine embryonic fibroblasts (MEFs) [32]. By contrast, the promoter regions of GAPDH, βactin, and Cnpy3 provided excellent negative controls since they were actively transcribed in MEFs and thus not subjected to H3K27me3-mediated silencing. Direct comparison of the three commercially available anti-H3K27me3 antibodies in miniChIP–qPCR highlighted the importance of performing a broad titration range in order to accurately assess antibody quality (Figure S2). We found that the H3K27me3 antibodies from Millipore and Abcam showed improved specificity and reduced signal-to-noise ratios compared to the Active Motif source. Based on these observations, the Abcam and Millipore anti-H3K27me3 were selected for their use in subsequent experiments [28,32].

**Figure 3:**
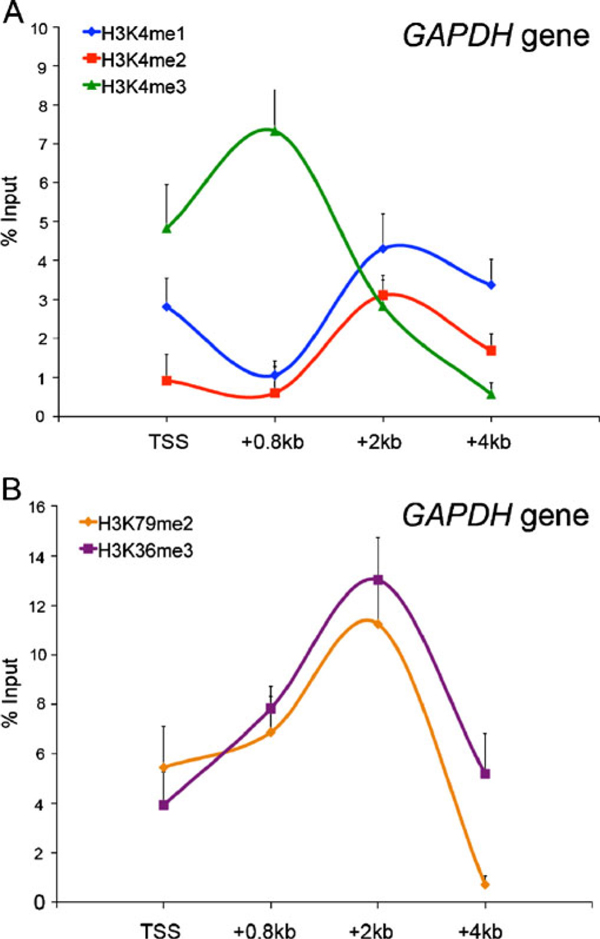
**The spatial distribution of H3K4me3 across the GAPDH gene**. Using antibodies specific for H3K4me1, H3K4me2, H3K4me3, H3K79me2, or H3K36me3, the enrichment levels (% input) of these different histone modifications were determined in MEFs at the GAPDH transcriptional start site (*TSS*) and gene body. **a** The distribution profiles of H3K4me1, H3K4me2, and H3K4me3 at GAPDH. **b** The distribution profiles of H3K79me2 and H3K36me3 at GAPDH. In both figures, the genomic region analyzed by qPCR is shown on the *x*-axis and is designated as kilobase (kb) distance from the TSS. *Error bars* represent the standard deviation (±SD) of three independent ChIP assays.

Another important parameter of miniChIP setup was the selection of genomic loci that accurately reported on reagent quality. Genomic loci that are associated with similar enrichment profiles of histone modifications regardless of cell type were specifically chosen for this analysis. Thus, for the assessment of activating histone modifications, we took advantage of the ubiquitously expressed housekeeping genes, GAPDH, Cnpy3, and βactin. For example, the GAPDH promoter and exon regions were associated with the activating histone modifications H3K4me3, H3K36me3, H3ac, and H3K79me2, as well as PolII. These histone modifications showed a distinct distribution pattern across the GAPDH gene consistent with previous genome-wide ChIP–chip and ChIP–seq analyses [16,35,36]. H3K4me3 enrichment was observed in close proximity to the transcriptional start sites (TSS) and characterized by a narrow trough roughly at the TSS with highest enrichment levels localized to either side (Figure 3a). By contrast, the mono- and di-methylation states of H3K4 showed highest enrichment up and downstream to the TSS and revealed an overall reciprocal enrichment pattern compared to H3K4me3. Two marks associated with transcriptional elongation, H3K79me2 and H3K36me3, revealed highest enrichment levels in the GAPDH gene body (Figure 3b). A similar trend was noted for the elongating polymerase II (PolII) using antibodies directed against the serine residue 5 of PolII (data not shown). While the GAPDH gene provided a reliable negative control for the assessment of H3K9me3 (and H3K27me3) silencing histone modifications, the selection of ubiquitous positive controls for H3K9me3 was more challenging. However, we have found that the p16^Ink4a^ and Pax5 promoters provide reliable controls in early passage MEFs (Figure S3).

To our knowledge, only two other laboratories have reported the use of miniaturized scaled ChIP assays in loci-specific and global microarray-based surveys [26,27]. The technical difficulties and multiple steps associated with this method could account for why many laboratories have failed to successfully implement this technology. Thus, steps such as sonication optimization, antibody titrations, and the analysis of appropriate genomic loci for antibody testing, as well as the use of high recovery, low retention plastics (Eppendorf tubes and filtered pipette tips) should be carefully monitored. Our successful implementation of miniChIP was also reliant on the use of strict molecular biology laboratory skills, faithful recapitulation of the method, use of calibrated and clean pipettes, careful pipetting techniques, correct storage of reagents, regular quality control testing, and maintenance of ultra clean workspaces.

### 3.2 Global MiniChIP–Chip Technology

MiniChIP–qPCR was highly effective at resolving small numbers of cells. However, this approach became limited in scope when performing experiments that aimed to identify histone modification profiles at numerous genomic loci or genome wide. This was, in part, explained by the low amounts of DNA recovered from 10,000 cells, which permitted the analysis of about 10 different genomic regions. The recent development of DNA amplification methods suited for low quantities of genomic DNA enabled the coupling of miniChIP assays to promoter tiling arrays in order to increase target identification. Most recently, we and others have described the use of whole genome amplification kits (Sigma WGA4) optimized for low concentrations of ChIP DNA and NimbleGen promoter tiling microarrays in order to develop miniaturized scaled ChIP–chip studies [26-28]. Our most recent study was based on the analysis of data obtained from 72 separate NimbleGen high density, 2.1 million feature promoter tiling arrays, thus enabling the investigation of five different histone modifications and PolII (6 ChIP conditions) in four primary cell types, each performed in triplicate [28]. In this section, we describe detailed computational methods and quality control steps that facilitated our investigation of epigenetic chromatin modifications during cell fate decisions of murine HSCs in bone marrow using miniChIP–chip.

The NimbleGen promoter tiling array platform comprised two-color arrays and were subject to non-biological variations including dye-bias resulting from different labeling efficiencies of the Cy3 and Cy5 dyes [37]. These variations lead to an intermixture of signals from enriched and unenriched samples and therefore caused an increase in background noise that hindered the identification of enriched regions. Additional variations resulted from the array processing itself, especially when the array number was too high to be processed in a single batch experiment. In our study, since the 72 arrays were processed in four separate batches, we anticipated differences in handling and use of multiple reagent lots. Such variations potentially affected the overall range of signals between arrays and therefore reduced sample comparability. In order to address these issues, we began our data analysis with an effective normalization strategy. For this purpose, a two-step normalization protocol was implemented (Figure 1(A, B)). In the first step, the raw data was normalized using the MA2C software designed especially for "within-array" normalization of NimbleGen ChIP–chip data (Figure 1(A)) [37,38]. The second step comprised a quantile normalization approach (Figure 1(B)) [36,39]. This method allowed for "between array" normalization, which aimed to enhance the comparability of signal ranges between replicate arrays by defining the final intensity of probes within a specific quantile as the average value of this quantile. Applying the above steps to the array data and subsequently averaging replicate intensities (Figure 1(C)), histone modification profiles for the four hematopoietic cell types were derived and subsequently investigated for their association with lineage commitment [28].

To confirm that this protocol produced reliable data and effectively eliminated any non-biological variations or technical artifacts, we performed exhaustive quality control confirmations following each normalization step [28]. Within array normalization was especially important when dealing with DNA oligonucleotide microarrays. This method enabled us to determine the extent by which dye-bias or other non-biological variations had influenced the raw data. Within array normalization was visually investigated using *M* versus *A* plots, where *M* was the difference in log intensity (*M* = log_2_(*R*/*G*); with *R* and *G* as the intensity of the red and green channel, respectively) and *A* was the average of the log intensity values ($A=\frac{1}{2}\log_{2}\left(R*G\right)$) for each probe [40]. In the absence of any dye-bias, the majority of the data, i.e., the unenriched probes, clustered around the horizontal line *M* = 0, without showing any dependence on the intensity *A *[37]. Deviations from this appearance as manifested by intensity shifts or rotations away from the horizontal line, were clear indications of non-biological artifacts to the data, and were corrected using proper data preprocessing. Accordingly, the MA plots provided a useful tool in estimating the need for specific data preparation steps as well as assess and fine-tune the performance of our normalization strategy (Figure 1(A)—QC step, Figure S4).

Following within-array normalization, differences in data range of the replicate arrays persisted. This was due to the hybridization process itself, which introduced a shift in the signal intensity causing the range of signals to be different between the replicate arrays. Therefore between-array normalization was employed using a quantile normalization approach. This method resulted in the scaling of all signals obtained from replicate arrays into the same data range. Inspecting and comparing the range of signals between the corresponding arrays using box and whisker plots directly tested the performance of this normalization approach (Figure 1(B)—QC step). Scatter plots and Pearson correlation estimates were performed between each pair of replicates before and after normalization (Figure 1(B)—QC step). Importantly, the quantile normalization strategy was not used for array data obtained from different cell types. The strategy removed differences in signals observed across cell types that resulted from biological changes. Instead, array comparability was tested through inspection of a panel of housekeeping genes, which showed highly similar enrichment profiles across the range of cell types studied (Bing Ren and Gary Hon, *personal communication*).

Upon completion of the normalization steps, significantly enriched regions within the normalized Log2 ChIP/input ratios against the background of the unenriched sample were determined (Figure 1(D)). In our study, the detection of enriched regions or "peaks" was performed using the NimbleScan software v2.1 in accordance with NimbleGen Systems standard operating procedures [28]. The input data represented the average of the normalized probe data obtained from triplicate arrays. NimbleScan peaks were determined as regions comprising at least four adjacent probes that showed a Log2 intensity above a cut-off value of 90% down to 15% of a theoretical maximum enrichment value. These regions were identified using a sliding window of 500 base pairs width. Following peak finding, the ratio data was permutated in order to calculate false discovery rates (FDR) for the observed peaks. To limit the drawing of conclusions from a dataset comprising of possible false positives, only the most stringent peaks were considered that showed a FDR ≤0.05. However, less stringent peaks, as indicated by 0.05 ≥ FDR ≤ 0.2, were included for some quality control analyses, and became useful when comparing individual loci. For example, we found that the lower stringent peaks provided insight into the degree of resolution differences between replicates due to hybridization and other array processing technical artifacts [28].

When mapping the identified NimbleScan peaks to annotation tracks, in our case to the NCBI gene tracks (Figure 1(E)), a large proportion of peaks were found in close proximity and thus matched to a number of different transcriptional start sites within the overlapping 11.2 kb (-8.2 kb to +3 kb) tiling regions. Therefore, we sought to obtain a unique association between single peaks and promoters in order to reduce the number of false positives. This approach enabled the allocation of each peak to a single promoter rather than linking a single peak across numerous promoters, and potentially leading to false positive and negative data. Since individual inspection for the unique linkage of peaks and promoters was not possible in our genome-scale study, we resolved this by linking each peak to the most proximate TSS within respective 11.2 kb tiled regions.

In addition, the number of peaks at enriched regions were often highly variable in that many promoters were found associated with a varying numbers of peaks, sometimes >15 peaks per promoter. However, close inspection of the enriched regions revealed that a high number of peaks was not indicative of an overall larger enriched region, since a single broad peak was often found to a span larger genomic region compared to the multiple smaller peaks. This phenomenon was likely caused by stochastic variances or processing related hybridization/labeling issues that introduced random intensity reductions in one or more probes within the enriched regions. The NimbleScan peak finding algorithm interpreted these gaps or signal reductions as unenriched regions. Therefore, a single enriched region was potentially split into a multitude of smaller adjacent peaks. Since we were unable to control for whether these differences in peak variance were biological or technical related, our analysis was based on present/absent peak calls rather than peak number in order to identify genes enriched for specific histone modifications. Thus, a promoter was defined as being enriched for a histone modification if it comprised at least one peak of FDR ≤0.05.

To further confirm the NimbleScan peaks, additional analyses of the NCBI-mapped data were performed. These included global evaluations of genomic distribution maps. Histograms were created in order to show the number of peaks detected at specific genomic distances linked to TSSs (Figure 1(E) QC). In our study, we identified the expected distribution of histone modifications, which confirmed previous studies [16,28]. We employed individual inspection of loci in order to visualize differences between replicates and to confirm the correct identification of bound regions (Figure 1(E) QC). The ideal targets for this analysis included well-characterized promoters for each of the given histone modifications including housekeeping genes. Genomic data were routinely confirmed for approximately 12 promoters using ChIP–qPCR and biologically independent samples (Figure 1(E) QC).

**Figure 4:**
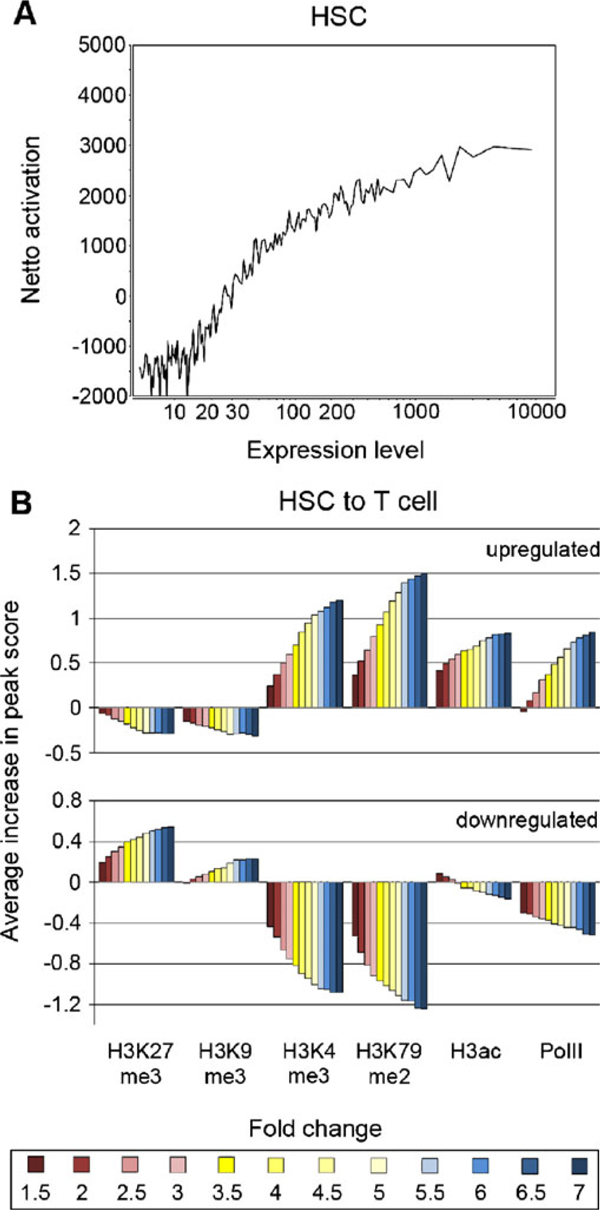
**Correlation of histone modifications and gene expression**. **a** Correlation of summarized net enrichment of five histone modifications (H3K4me3, H3ac, H3K79me2, H3K27me3, and H3K9me3) and PolII (*y*-axis) at genes with expression levels of the corresponding ~23,000 genes (*x*-axis). Arbitrary values of net enrichment were calculated by summing the individual features (enriched basepair (bp) area multiplied by highest peak score) of all histone modifications, while considering the effect of silencing histone modifications (H3K27me3 and H3K9me3) as a negative activation, and subsequently normalizing for the number of activating and silencing histone modifications present at each promoter. Genes were clustered into bins of 100 genes each. **b** Increase of Log_2_ enrichment scores (*y*-axis) upon up- or downregulation of gene expression levels between murine hematopoietic stem cells (*HSC*) and splenic CD4^+^ T cells (*T cell*) for five histone modifications (H3K4me3, H3ac, H3K79me2, H3K27me3, and H3K9me3) and PolII (*x*-axis). A total of 12 different fold change categories for up- and downregulation of gene expression is shown (fold changes of 1.5, 2, 2.5, 3, 3.5, 4, 4.5, 5, 5.5, 6, 6.5, and 7). The increase in Log_2_ peak scores for the histone modifications and PolII was averaged at gene promoters belonging to each category of fold change in gene expression (*y*-axis).

The resultant processed miniChIP–chip data was analyzed according to the requirements of an individual study design. An initial analysis included the incorporation of global gene expression data in order to investigate the effect of histone modifications on gene activation, silencing and poising. Indeed, our previous analyses revealed a positive correlation trend for modifications reported as activating in the literature, while the opposite negative correlation was true for silencing histone modifications [28]. In Figure 4, this analysis was developed further in order to determine the combinatorial effect of the investigated histone modifications on gene expression. In this study design, we defined an arbitrary value to describe the combined effect of activating and silencing histone modifications and PolII on gene promoters. We derived "net enrichment" by calculating for each peak the bound area multiplied by peak score and then summing the results for all peaks and conditions, while assigning negative signs to peaks of silencing histone modifications. Thus we observed high net values for genes exclusively bound by activating modifications, negative net values for genes bound only by silencing modifications and intermediate values for genes with combinatorial histone modification profiles. When net enrichment at promoters was related to gene expression, a high degree of the expected correlation was demonstrated (Figure 4a). Accordingly, gene expression was positively correlated to net enrichment while the opposite negative correlation was observed for genes lacking expression or showing expression at low levels. To investigate these trends in greater detail, the changes in histone modification peak scores were analyzed in the context of up- or downregulation of gene expression. For this purpose, the average change in peak score of the five histone modifications (H3K4me3, H3ac, H3K79me2, H3K27me3, and H3K9me3) and PolII over a range of differential gene expression changes between HSCs and CD4^+^ T cells was investigated (Figure 4b). Our analysis revealed a strong positive correlation with regard to gene upregulation and peak scores of the activating histone modifications and PolII. Likewise, a correlative decrease of the silencing histone modification peak scores was observed at the upregulated genes confirming the net enrichment trends (Figure 4a and 4b). As expected, the opposite correlation trends where observed for the downregulated genes. Therefore, our data revealed that the change in peak score values was highly proportional to the degree of up- or downregulation of gene expression.

## 4 Summary

The miniChIP–chip approaches described here can be utilized to identify the chromatin states of primary cell types that are present as both rare and abundant populations in tissues and organs across many model organisms. Recent developments in epigenetic-based technologies, including miniChIP–chip, will continue to pave the way in expanding our knowledge on the mechanisms of epigenetic inheritance, gene transcription, and genomic function in developmental processes and tissue homeostasis. Epigenetic resolution of rare cell populations responsible for maintaining a range of pathological conditions including inflammation and cancer is becoming an achievable goal for most laboratories. These methods will help to uncover novel targets in complex biology and disease.

## 5 Protocols

### 5.1 Miniaturized Chromatin Immunoprecipitation (MiniChIP) Assay Based on 10,000 Cells

Notes: It is critical that each reaction volume represents 10,000 cells otherwise the assay will not be reproducible or consistent. Low retention barrier tips and Eppendorf tubes must be used in all steps. Do not pipette up and down when adding solutions to tubes. The procedure must be conducted in a time efficient manner without long breaks between steps. Working stocks of buffers (e.g., 1 M Tris, 0.5 M EDTA, 5 M NaCl, and 10% SDS) should be autoclaved and stored at room temperature. The ChIP buffers should be 0.2 μM filtered and stored at 4°C, and can be conveniently made in sterile 50-ml falcon tubes. All steps must be performed on ice or cold room unless otherwise stated.

#### 5.1.1 A. Sonication Optimization

Formaldehyde crosslinking and cell lysis

1. Sort 50,000 cells into 1 ml of DMEM + 10%FBS in an Eppendorf tube. This number of cells will allow for five sonication conditions comprising 10,000 cells. For visualization of sheared DNA from 10,000 cells on agarose gels, 3 × 50,000 cell replicates will be required. We recommend using a primary cell population that is abundant and easy to isolate using FACS, e.g., CD4^+^ T cells from mouse spleen or a passaged cell line.

2. Add 2.7 μl of 37% formaldehyde (0.1% final concentration) and invert tubes immediately two to three times to ensure complete mixing. Incubate at room temperature for 10 min with occasional manual agitation.

3. Pellet cells by centrifuging at 2,500 rpm (300 rcf) for 10 min at 4°C.

4. Transfer tubes to ice and remove supernatant.

5. Add 1 ml of ice-cold HBSS containing protease inhibitor cocktail (HBSS+PIC), and invert the tubes three to four times to wash cells.

6. Pellet cells by centrifuging at 2,500 rpm (300 rcf) for 10 min at 4°C.

7. Transfer tubes to ice and remove supernatant.

8. For 50,000 cells, add 125 μl of Lysis buffer and 1.25 μl of PIC (100× stock) so that each 10,000 cell quantity will be lysed with 25 μl. Do not pipette up and down or vortex, instead manually agitate the bottom of the Eppendorf tubes, and allow bubbles to form.

9. Incubate on ice for 5 min to ensure complete lysis.

10. Add 375 μl of HBBS+PIC (5 × 75 ul) to tubes and mix by gentle inversion four to six times. If the SDS forms a white cloudy precipitate, place the tubes at room temperature for about 2–3 min and mix by gently tapping the tube until precipitate dissolves. Place directly on ice and proceed to step 11 immediately. It is important that aliquots are prepared without SDS precipitation.

11. Aliquot 100 μl (25 μl lysis buffer + 75 μl HBSS+PIC containing 10,000 cell equivalents) of cell lysate into new Eppendorf tubes (15 in total).

Sonication optimization

1. Place the tubes into a sonicator water bath unit (Diagenode) that contains prechilled water to the recommended volume, and a 2 cm thick layer of ice for cooling. Perform the sonication using the 30-s on/off cycle program and a high setting according to the manufacturer's instructions. For each cycle, three tubes each comprising a cell lysate equivalent of 10,000 cells will be processed.

Tube 1: one cycle

Tube 2: three cycles

Tube 3: five cycles

Tube 4: seven cycles

Tube 5: 10 cycles

2. Replenish the 2 cm ice layer to maintain the temperature at 2–8°C during sonication.

3. Remove tubes from the sonicator once the desired number of cycles has been reached, and place back on ice.

4. Collect the soluble chromatin by centrifugation at 13,000 rpm (17,000 rcf) for 10 min at 4°C. Note that a tight white pellet will be present following centrifugation. This will comprise precipitated SDS, which should be avoided when removing the supernatants. Transfer the supernatants to new Eppendorf tubes and pool according to cycle number. The pooled volume for each cycle condition will be 300 μl and there will be five tubes in total.

5. Add 10 μl of 5 M NaCl and 1 μl of 20 mg/ml of Proteinase K to each tube.

6. Incubate with mixing at 1,300 rpm 68°C for 2 h using a thermomixer (Eppendorf).

DNA purification

1. Briefly centrifuge tubes to remove sample from lid and walls of the tube.

2. Add 300 μl of phenol/chloroform/isoamylalcohol and mix by vortexing for ~3 s.

3. Centrifuge samples for 10 min at 13,000 rpm (17,000 rcf) at room temperature.

4. Transfer the upper aqueous phase (300 μl) into new a tube.

5. Add 30 μl of 3 M sodium acetate (pH 5.2), 2 μl of linear acrylamide (5 μg/μl stock) and 2 μl of glycogen (5 μg/μl stock) to the supernatant, and mix by manual agitation of tubes.

6. Add 800 μl of 97% ethanol (stored at -20°C). Mix tubes by inversion four to six times and incubate for ~16 h at -20°C or 1 h at -80°C.

7. Centrifuge the tubes at 13,000 rpm (17,000 rcf) for 20 min at 4°C to collect the precipitated DNA.

8. Remove the supernatant. Add 1 ml of 70% ethanol (stored at -20**°**C) and invert the tubes four to six times.

9. Centrifuge the tubes at 13,000 rpm (17,000 rcf) for 20 min at 4°C.

10. Remove the supernatant.

11. Air dry the DNA pellet and resuspend in 20 μl T_10_E_0.1_ (10 mM Tris–HCl pH 8.0, 0.1 mM EDTA pH 8.0) buffer. Store the DNA samples at -20°C.

Agarose gel electrophoresis

**Figure 5:**
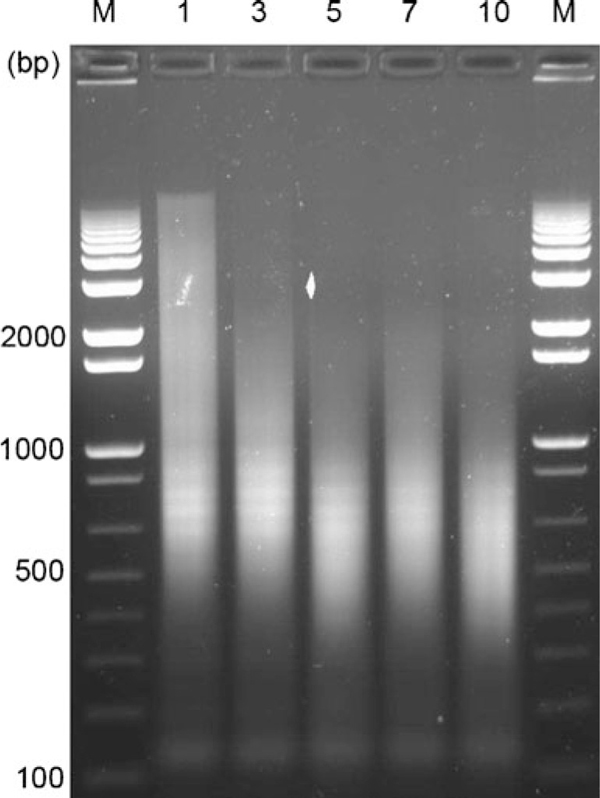
Visualization of sonication of chromatin obtained from 10,000 cells using 1% agarose gels

Note: five, seven, and 10 cycles gave fragments ranging from 200–1,000 bp. In subsequent miniChIP experiments with 10,000 cells, five cycles were used. Although seven and 10 cycles provided a good size range, there was risk of over sonication and chromatin degradation using greater cycle numbers. This image is also shown in supplemental Figure 1.

1. Run the entire sample (20 μl) onto a 1% agarose gel in order to visualize shearing efficiency. See Figure 5 below 

This protocol is designed for 10,000 cells per ChIP reaction. A single tube can be processed, starting with the formaldehyde crosslinking of 10,000 cells with 0.1% FA in 1 ml of DMEM + 10%FBS as described in Materials and Methods. However, typical miniChIP experiments comprise different antibody reactions. Therefore, we have chosen to describe a method that allows for three antibody conditions.

Formaldehyde crosslinking and cell lysis

1. Sort 30,000 cells into 1 ml of DMEM + 10%FBS into an Eppendorf tube. This will allow for three antibody reactions that each comprise 10,000 cells.

2. Add 2.7 μl of 37% formaldehyde (0.1% final concentration) and invert tubes immediately two to three times to ensure complete mixing. Incubate at room temperature for 10 min with occasional manual agitation.

3. Pellet cells by centrifuging at 2,500 rpm (300 rcf) for 10 min at 4°C.

4. Transfer tube to ice and remove supernatant.

5. Add 1 ml of ice-cold HBSS containing protease inhibitor cocktail (HBSS+PIC), and invert the tube three to four times to wash cells.

6. Pellet cells by centrifuging at 2,500 rpm (300 rcf) for 10 min at 4°C. Following centrifugation a tiny pellet will be barely visible.

7. Transfer tube to ice and remove supernatant. At this point, the cell pellet can be snap frozen on dry ice or liquid nitrogen and stored indefinitely at -80°C.

8. For 30,000 cells, add 75 μl of Lysis buffer and 0.75 μl of PIC (100× stock) such that each 10,000 cell quantity will be lysed with 25 μl. Do not pipette up and down, instead manually agitate the bottom of the Eppendorf tube, and allow bubbles to form.

9. Incubate on ice for 5 min to ensure complete lysis.

10. Add 225 μl of HBSS+PIC to tube and mix by gently inverting the tube four to six times and place back on ice. If the SDS forms a white cloudy precipitate, place the tube at room temperature for about 2–3 min and mix by gently tapping the tube until precipitate dissolves. Place directly on ice and proceed to step 11 immediately. It is important that aliquots are prepared without SDS precipitation.

11. Aliquot 100 μl (25 μl lysis buffer + 75 μl HBSS+PIC that contains 10,000 cell equivalents) of cell lysate into three new Eppendorf tubes.

Sonication

1. Sonicate the samples for five cycles as described above in Part A—Sonication Optimization. Perform five cycles of sonication using the 30-s on/off cycle setting.

2. Collect the soluble chromatin by centrifugation at 13,000 rpm (17,000 rcf) for 10 min at 4°C. Transfer the supernatants to a single Eppendorf tube (300 μl), while avoiding the small SDS pellets. Pooling of supernatants following sonication eliminates shearing variation and noise.

3. Add 330 μl (the extra 30 μl allows for the 10% input control and pipetting error) of ice-cold 2× RIPA Buffer (twofold dilution), and 6 μl of PIC (100× stock). Mix by tube inversion three to four times. The final SDS concentration will be ~0.1%, which is suitable for antibody immunoprecipitation.

4. Transfer 20 μl of the chromatin into a new Eppendorf tube. This is used as the input chromatin control and can be stored on ice until DNA purification.

Antibody immunoprecipitation

1. Aliquot 200 μl of the diluted chromatin into three new Eppendorf tubes.

2. Add the antibodies. The amounts below were empirically determined in antibody titration experiments (Figures 2, S2, and S3). Two micrograms of normal rabbit IgG provides a control for the 2 μg of anti-H3K27me3 antibody and 0.25 μg of anti-H3K4me3 used in this experiment.

Tube 1. Anti-H3K4me3 (Abcam ab8580) 0.25 μg (0.25 μl of 1 μg/μl stock)

Tube 2. Anti-H3K27me3 (Millipore 07-499) 2 μg (2 μl of 1 μg/μl stock)

Tube 3. Normal Rabbit IgG (Millipore 12-370) 2 μg (2 μl of 1 μg/μl stock)

3. Incubate for 2 h at 4°C (cold room) on a rotator device with a setting of 200 rpm. This step can be carried out up to 16 h depending on the efficiency of the antibody. However, prolonged incubation times can increase non-specific antibody binding and chromatin degradation, and is therefore not encouraged.

Antibody–bead immunoprecipitation

1. For 3 miniChIP reactions, pipette 35 μl (the extra 5 μl allows for pipetting error) of well-suspended Dynabeads^®^ protein A stock solution into a new Eppendorf tube on ice.

2. Add 200 μl of ice-cold 1× RIPA Buffer and mix well by tapping the tube, place the tube in the magnetic holder (placed directly onto ice), allow beads to be captured, and remove the buffer with a pipette.

3. Repeat the wash with another 200 μl of ice-cold 1× RIPA buffer. Ensure the beads are fully resuspended during this wash step.

4. Capture the beads on the magnet, remove the buffer, and add a final volume of 35 μl of ice-cold 1× RIPA buffer.

5. Add 10 μl of the Dynabeads^®^ protein A to each tube. Ensure that the beads remain resuspended by frequently agitating the tube.

6. Incubate for 2 h at 4°C (cold room) on a rotator device with a setting of 200 rpm.

Antibody–bead complex washes and elution

Washing of the antibody–beads complexes are performed according to the method outlined by Dahl and Collas [41]. Washes should be performed in a 4°C cold room.

1. Centrifuge the tubes (2 s pulse) to remove sample from the lid of tubes.

2. Place tubes onto chilled magnet rack (placed directly onto ice), allowing beads to adhere and remove supernatant.

3. Remove tubes from magnet rack, add 100 μl of ice-cold 1× RIPA buffer. Gently tap tube to resuspend the beads, and place onto rotator device.

4. Rotate tubes at 200 rpm for 4 min at 4°C.

5. Repeat steps 2. and 3. twice.

6. Centrifuge the tubes (2 s pulse), place them onto the magnet rack and remove the supernatant.

7. Remove tubes from magnet rack and place on ice, add 100 μl of ice-cold TE Buffer. Gently tap tube to help resuspend the beads, and place onto rotator device.

8. Rotate tubes at 200 rpm for 4 min at 4°C.

9. Centrifuge the tubes (2 s pulse), place onto the magnet rack and remove the supernatant.

10. For complex elution, add 300 μl of Elution Buffer (20 mM Tris pH 7.5/5 mM EDTA/50 mM NaCl/1% SDS/50 μg/ml proteinase K) to the bead pellets and incubate for 2–3 h at 65°C using a shaking Eppendorf Thermomixer (1,300 rpm).

DNA purification

1. Briefly centrifuge tubes to remove sample from lid and walls of the tube.

2. Place tubes onto the magnet rack and transfer the supernatant containing the eluted complexes into new tubes.

3. Add 300 μl of phenol/chloroform/isoamylalcohol and mix by vortexing for ~3 s.

4. Centrifuge samples for 10 min at 13,000 rpm (17,000 rcf) at room temperature.

5. Transfer the upper aqueous phase (300 μl) into new a tube.

6. Add 30 μl of 3 M sodium acetate (pH 5.2), 2 μl of linear acrylamide (5 μg/μl stock) and 2 μl of glycogen (5 μg/μl stock) to the supernatant, and mix by manual agitation of tubes.

7. Add 800 μl of 97% ethanol (stored at -20°C). Mix tubes by inversion four to six times and incubate for ~16 h at -20°C or 1 h at -80°C.

8. Centrifuge the tubes at 13,000 rpm (17,000 rcf) for 20 min at 4°C to collect the precipitated DNA.

9. Remove the supernatant. Add 1 ml of 70% ethanol (stored at -20**°**C) and invert the tubes four to six times.

10. Centrifuge the tubes at 13,000 rpm (17,000 rcf) for 20 min at 4°C.

11. Remove the supernatant.

12. Air dry the DNA pellet and resuspend in 10 μl T_10_E_0.1_ (10 mM Tris–HCl pH 8.0, 0.1 mM EDTA pH 8.0) buffer. Store the DNA samples at -20°C.

Real-time quantitative PCR analysis

The low concentration of DNA recovered from miniChIP reactions with 10,000 cells allows for qPCR analysis of ~8 genomic regions to be performed in triplicate qPCR reactions. Therefore, this assay provides enough DNA for a total of 24 individual qPCR reactions.

The individual qPCR reaction volumes are 20 μl, and two different master mixes are prepared. The ChIP DNA mastermix is added to 96-well PCR plates prior to the SYBR green master mix in order to minimize cross contamination.

1. ChIP DNA or input DNA master mix

(a) Thaw DNA samples on ice.

(b) Prepare the ChIP DNA or input master mixes by adding 190 μl sterile milliQ water (mqH_2_O) to each of the tubes containing 10 μl of DNA (200 μl total volume), mix by manual agitation, and centrifuge briefly (2 s pulse), and place on ice.

(c) Since the SYBR green mastermix will account for 12 μl in a 20 μl qPCR reaction volume (see below), aliquot 8 μl of each DNA sample to respective wells in a PCR 96-well plate. Therefore, in each final qPCR reaction, ~0.5 μl of the ChIP DNA sample will be analyzed if 24 reactions are performed. There should be 8 μl remaining to accommodate variations in pipetting.

2. SYBR green/primer master mix

(a) Prepare SYBR green/primer master mixes for the number of genomic regions analyzed. For 20 μl qPCR reaction volumes, the mastermix will comprise SYBR Green Master Mix (2×) 10 μL, forward primer (10 mM stock) 1 μL and reverse primer (10 mM stock) 1 μl.

(b) Aliquot 12 μl into appropriate wells that already contain 8 μl of the ChIP or input DNA samples.

3. qPCR analysis

(a) Perform real-time PCR of the samples using a 40-cycle program.

(b) Acquire the data using the real-time PCR data acquisition program. Most programs require that you manually adjust the threshold value to the linear range of the real-time PCR curve. Keep this value constant when directly comparing experiments.

(c) Export the data into Excel spreadsheets.

(d) Calculate the amount of precipitated DNA relative to input as % (ChIP/total input) = 2^[(*C*_t_(ChIP) - *C*_t_(input) × DF)] × 100%. At least three biological independent miniChIP experiments should be analyzed in triplicate qPCR reactions for each genomic region investigated.

(e) The SYBR qPCR detection method is based on using equally efficient primer sets. Prior testing of primer sets on input DNA or genomic DNA in a standard curve experiment is required. Similar *C*_t_ values should be obtained across a concentration range of 0.1 ng to 20 ng DNA. Melting curve analysis should reveal the presence of a single amplicon and the absence of primer dimers.

Promoter tiling microarray analysis

1. Library preparation and quality control analysis

(a) Thaw miniChIP DNA or input DNA samples on ice.

(b) Transfer the DNA samples (10 μL) into 0.2 ml PCR tubes.

(c) Conduct amplification of the DNA samples in 0.2 ml PCR tubes with the WGA4 GenomePlex Whole Genome Amplification Kit (Sigma Aldrich) according to the manufacturer's instructions. Note that the cell lysis and DNA fragmentation steps will be omitted and the protocol will start at Step 6 in the WGA4 procedure. A matching number of input and ChIP DNA samples are amplified to cover the number of ChIP DNA samples on the arrays.

(d) Purify the amplified DNA using the QIAquick PCR purification kit (Qiagen, catalogue #28104) according to the manufacturer's instructions.

(e) Perform qualitative and quantitative assessment of the amplified DNA samples using 1% agarose gels and NanoDrop Spectrophotometer, respectively. Typically, the WGA4 kit leads to the production of ~10 μg of DNA with an average size of 500–2,000 bp suitable for processing on NimbleGen microarrays.

(f) Perform qPCR surveys of genomic regions with the expected enrichments for the cell types and histone modifications examined. An example is provided in [28].

2. Array processing

1. Submit the amplified ChIP samples to NimbleGen Systems, Iceland for processing according to their standard protocols. The facility will perform the labeling, hybridization, and scanning using the HD2 Hx1 promoter tiling arrays (HD 2.1 M array) that span -8.2 kb to +3 kb around TSS of ~24,000 annotated genes.

3. Array normalization

1. Apply within-array normalization to single arrays by applying using the MA2C software [37,38] or equivalent within-array normalizations tools.

2. Apply quantile normalization to replicate arrays in order to facilitate between-array comparability and use the average value of each quantile as the final intensity for corresponding probes.

4. Identification of bound regions

1. Combine replicate arrays by averaging the Log_2_ enrichment values of each probe within the set of replicates.

2. Identify significantly enriched regions (peaks) using the peak finding algorithms as provided by the NimbleScan software according to NimbleGen standard protocols.

3. Annotate peaks with a false discovery rate ≤0.05 by mapping them to the NCBI gene tracks using the NimbleScan software and a range of 8.2 kb downstream to 3 kb upstream relative to each transcriptional start site.

4. Enforce unique associations between peaks and TSSs by linking each peak only to the most proximate TSS present in the annotated 11.2 kb region.

5. Quality control analysis

Possible quality control analyses can include the following:

1. Confirm within array normalization by deriving MA plots before and after normalization and check for resolution of any dye-bias related variations observed in the raw data.

2. Investigate the range of signals between replicates using bars and whisker plots in order to validate the quantile normalization step.

3. Validate the overall normalization strategy in terms of comparability between replicates by generating scatter plots and Pearson correlation coefficients for each pair of replicates. Use a sufficient sized set of probes to reduce biasing of the results.

4. Perform peak finding for single arrays before and after within array normalization as well as before and after quantile normalization, and compare the total base pair range occupied by peaks found with a FDR of ≤0.05 between the replicate arrays and different normalization steps. Total range of enrichment should remain in a comparable range for replicates and before and after normalization. The resultant normalization should result in a convergence of peak numbers between replicate arrays. Outsiders could represent failed ChIP assays or array processing.

5. Investigate distribution of peaks within the 11.2 kb region by plotting histograms of number of peaks against distance from TSS. Replicate should show highly similar distribution patterns also comparable with literature.

6. After mapping the peaks to gene tracks (e.g., NCBI), estimate the percentage of genes found enriched for each of the different histone modifications and refer to literature to confirm range of occupancy.

7. Investigate individual gene loci and compare normalized arrays and peaks to ChIP–qPCR results.

8. If gene expression data is included in the data, validate expected function of histone modifications (gene activating/silencing) using correlation analysis of genes expression values, peaks scores or enriched base pair range of promoters. Positive correlation should be observed for activating histone modification; silencing histone modifications should show a negative correlation with gene expression.

**Table:** 

*ChIP assay buffers*	
**1**× **RIPA Buffer (50 ml)**	
Final concentration:	Volume of stock buffers:
10 mM Tris–HCl, pH 7.5	500 μl of 1 M
1 mM EDTA	100 μl of 0.5 M
1% Triton X-100	5 ml of 10%
0.1% SDS	500 μl of 10%
0.1% Nadeoxycholate	500 μl of 10%
100 mM NaCl	1 ml of 5 M
Water	42.4 ml
**2**× **RIPA Buffer (50 ml)**	
20 mM Tris–HCl, pH 7.5	1 ml of 1 M
2 mM EDTA	200 μl of 0.5 M
2% Triton X-100	10 ml of 10%
0.1% SDS	500 μl of 10%
0.2% Nadeoxycholate	1 ml of 10%
200 mM NaCl	2 ml of 5 M
Water	35.3 ml
**Lysis Buffer (10 ml)**	
50 mM Tris–HCl, pH 8.0	500 μl of 1 M
10 mM EDTA	200 μl of 0.5 M
1% SDS	1 ml of 10%
Water	8.3 ml
**TE Buffer (50 ml)**	
10 mM Tris–HCl, pH 8.0	500 μl of 1 M
10 mM EDTA	1 ml of 0.5 M
Water	48.5 ml
**Elution Buffer (50 ml)**	
20 mM Tris–HCl, pH 7.5	1 ml of 1 M
5 mM EDTA	500 μl of 0.5 M
50 mM NaCl	500 μl of 5 M
Water	48 ml

Containing 1% SDS and 50 μg/ml proteinase K prior to use

All buffers must be 0.2 uM filtered sterilized, and are based on [41]

## Supplementary Material

Additional file 1 Click here for file
