# Supplementary material for: A Method to Study the Epigenetic Chromatin States of Rare Hematopoietic Stem and Progenitor Cells; MiniChIP–Chip
Source: Biol Proced Online. 2010 May 15;12:1–17. doi: 10.1007/s12575-010-9031-y (PMC3396287; doi:10.1007/s12575-010-9031-y)
Supplement: Additional file 1 [file 1480-9222-12-1-9031-S1.doc]

Supplemental Data for “A method to study the epigenetic chromatin states of rare hematopoietic stem and progenitor cells; miniChIP-chip ”

Holger Weishaupt^1^ and Joanne L. Attema^1*^

^1^Immunology Unit, Institute for Experimental Medical Science, BMC D14, Lund University, 221 84 Lund, Sweden

^*^Corresponding author:

Joanne Attema PhD

Lund University

Institute for Experimental Medical Science

Immunology Unit, BMC D14

221 84 Lund

Sweden

Phone: +46 46 222 3338

Fax: + 46 46 222 4218

E-mail: Joanne.Attema@med.lu.se

**Contents:**

4 Supplemental Figures

1 Supplemental Table

Supplemental Figure 1. (*Weishaupt and Attema*)

**
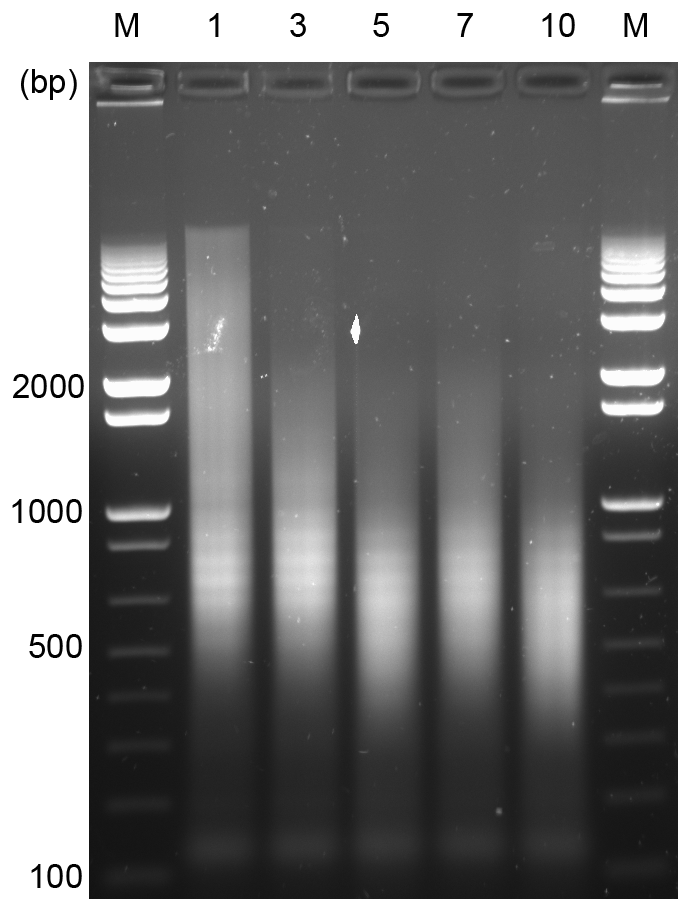
**

**Supplemental Figure S1. Visualization of sonication of chromatin obtained from 10,000 cells using 1% agarose gels.** 30 sec on/off cycles were tested with chromatin obtained from 10,000 cells in 100ul volumes over a range of 1, 3, 5, 7 and 10 cycles using a Diagenode Bioruptor. In order to visualize the purified genomic DNA on 1% agarose gels, 3 replicates were processed in parallel for each cycle condition tested, and subsequently pooled prior to the reversal of crosslinking protein digestion step and DNA recovery using phenol/chloroform extraction and ethanol precipitation (see Upstate/Millipore standard ChIP method, cat no. 17-295). Five on/off cycles were routinely used for the adequate sonication of chromatin obtained from 10,000 cells. This resulted in generation of chromatin fragments sizes ranging 300 -1000 base pairs (bp). The 1kb plus DNA ladder (Invitrogen) was used as a size reference (M).

Supplemental Figure 2. (*Weishaupt and Attema*)

**
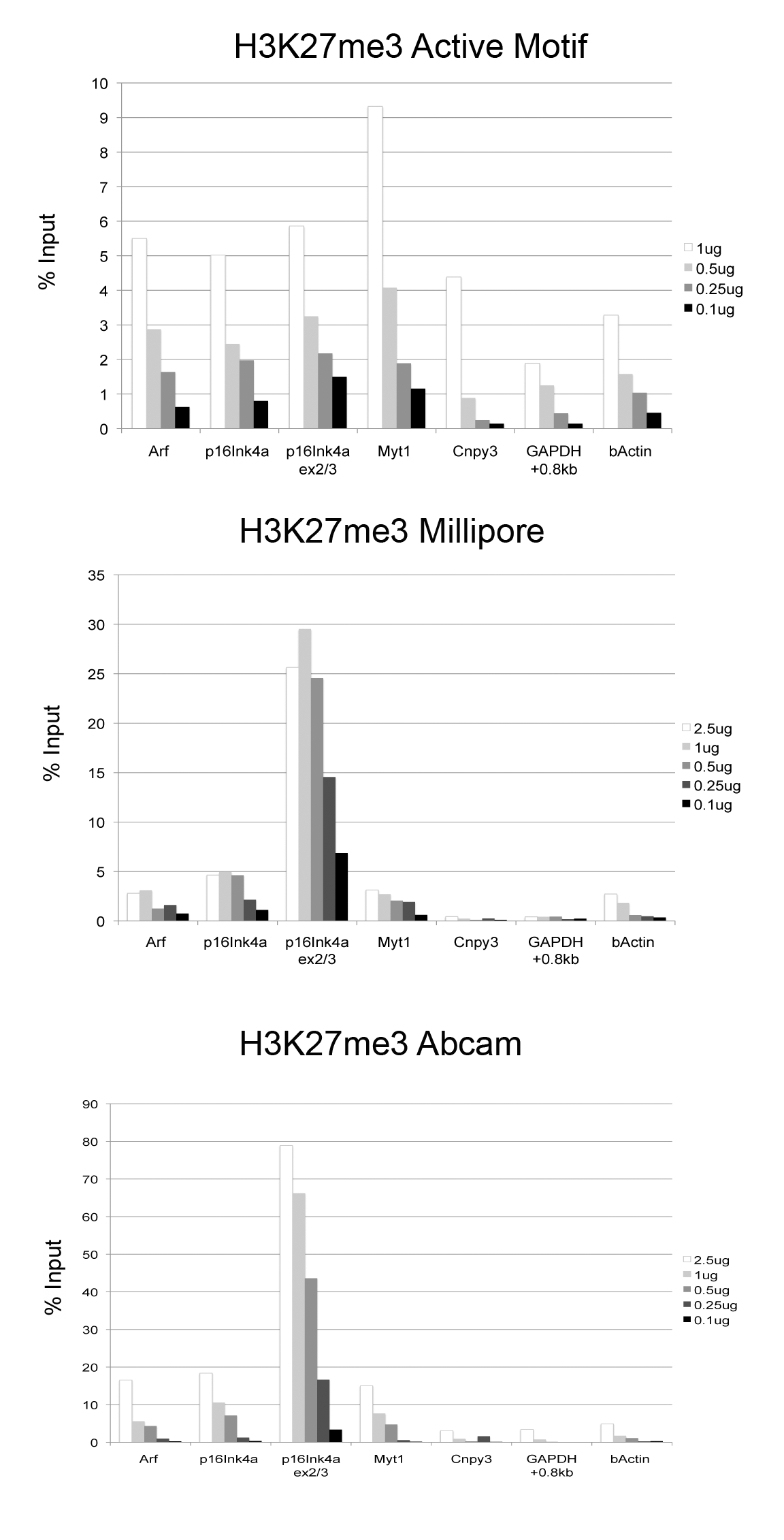
**

**Supplemental Figure S2. Quality assessment of anti-H3K27me3 antibodies in miniChIP assays.** Comparative analysis of anti-H3K27me3 antibody in miniChIP assays sourced from three different commercial companies, Active Motif AM 174 (top graph), Millipore 07-499 (middle graph) and Abcam ab6002 (bottom graph). The genomic regions in early passage MEFs analyzed by quantitative realtime PCR is shown on the x-axis, include the promoter and exon 2/3 regions of the p16^ink4a^ gene, and the TSSs of Myt1, Cnpy3, GAPDH and βactin. A range of antibody concentrations was tested as indicated in the figure legends.

Supplemental Figure 3. (*Weishaupt and Attema.*)

**
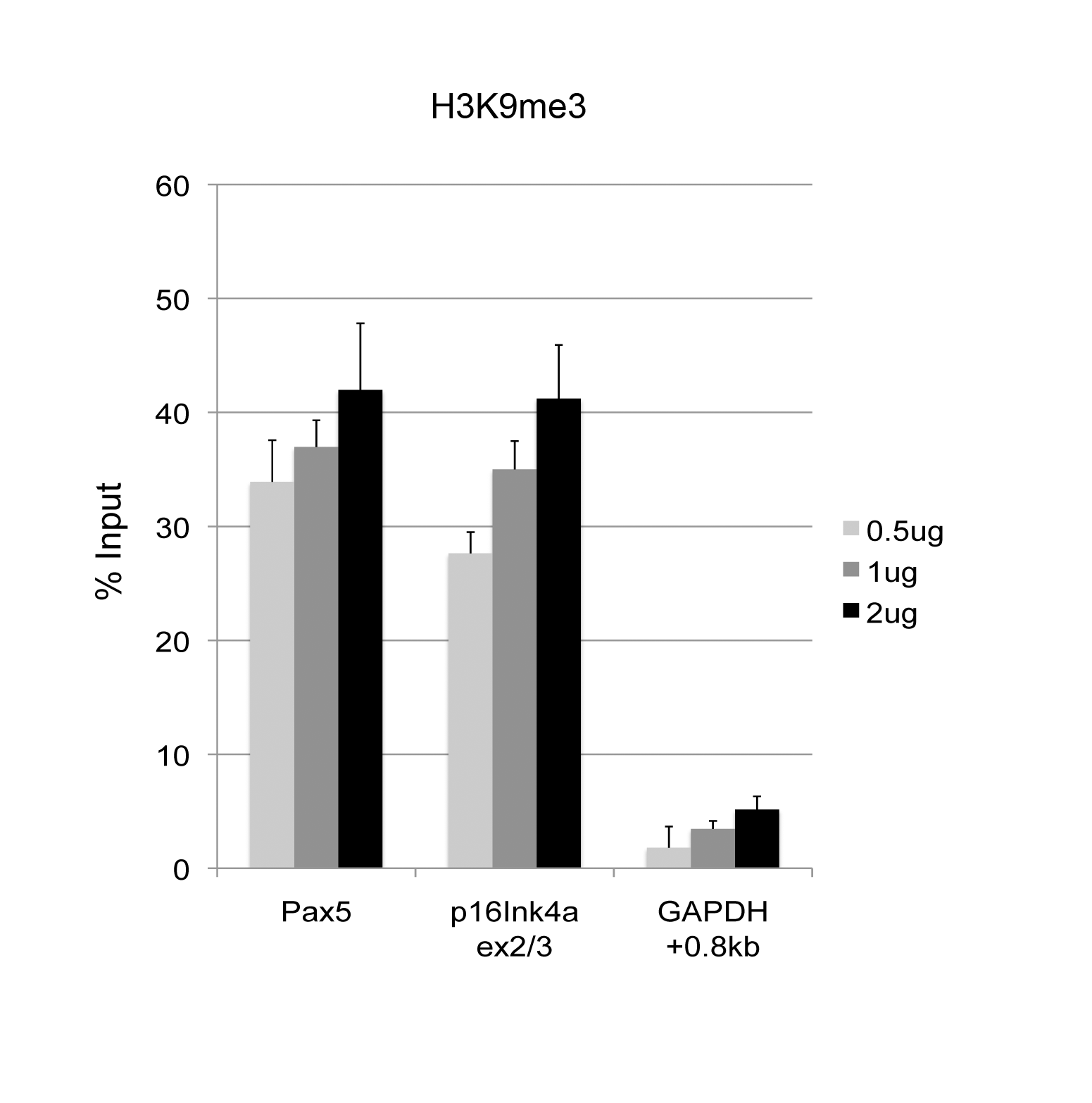
**

**Supplemental Figure S3. Performance of an anti-H3K9me3 antibody in miniChIP assays as assessed by robust target promoters, Pax5 and p16^ink4a^.** Assessment of the anti-H3K9me3 antibody (Abcam ab8898) in early passage MEFs at the Pax5, p16^ink4a^ and GAPDH TSSs using 0.5, 1 and 2ug of antibody. GAPDH served as a negative control whereas Pax5 and p16^ink4a^ are robust positive controls. Error bars represent the standard deviation (±SD) of 3 independent ChIP assays.

Supplemental Figure 4. (*Weishaupt and Attema.*)

**
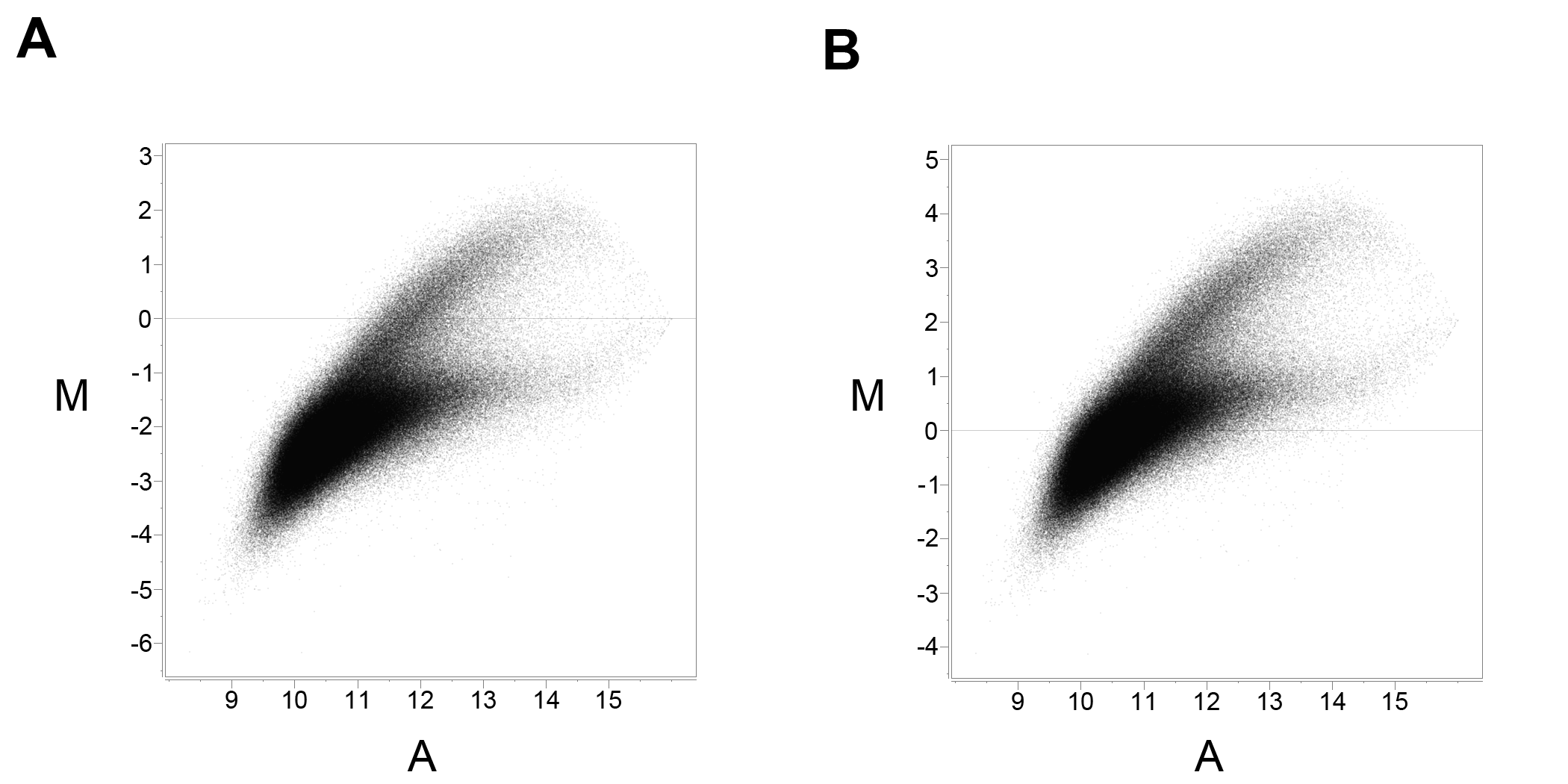
**

**Supplemental Figure S4. MA plots as a tool to assess data quality and performance of data preparation steps.** Data from the red and green channels of a two color array are depicted as *M* versus *A* plot, where *M* is the difference in log intensity (
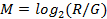
; with R and G as the intensity of the red and green channel, respectively) and *A* is the average of the log intensity values (
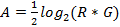
), as presented in (1). In the absence of any dye-bias, the majority of the data, i.e. the unenriched probes, should cluster around the horizontal line M = 0, without showing any dependence on the intensity A (2) (**A**) MA plot presenting the raw probe intensities of an arbitrary ChIP-chip two color array. The bulk of the unenriched probes falls clearly below M = 0 and the data also shows a strong dependence on A, thereby indicating the need for further data preprocessing. (**B**) MA plot after Bi-weight mean correction as performed by the NimbleScan software provided by NimbleGen. The unenriched data now clusters around M = 0, but there is still a strong dependence between M and A values implying that the dye bias persists and further normalization is required.

Supplemental Table S1 (*Weishaupt and Attema*)

| Genomic region | Forward Primer | Reverse Primer |
| --- | --- | --- |
| GAPDH TSS | TCCCCTCCCCCTATCAGTTC | GACCCGCCTCATTTTTGAAA |
| GAPDH +0.8kb | GCGGCCCGGAGTCTTAAG | GGATTACGGGATGGGTCTGA |
| GAPDH +2kb | GGGCAAGCAATCACCTCTTG | GGCCTGGCAGGGCTTTTA |
| GAPDH +4kb | GAGCCCTCCCTACTCTCTTGAAT | ACACCGCATTAAAACCAAGGA |
| βactin exon2 | CCACAGCTGAGAGGGAAATC | CTTCTCCAGGGAGGAAGAGG |
| Cnpy3 TSS | CCCGCTGCCTCTTATTTCCTTTG | CACCCAGTCGGTCTCCTCAG |
| Pax5 TSS | ATGGGAGTTTGTTTTCCTGTGT | AGTGATGTTTGGCCTAATCCTG |
| Myt-1 TSS | TGCCACCGCTGCTAATGAG | TGCGAACTCCTAAGCCAGCTA |
| p19-Arf TSS | AAAACCCTCTCTTGGAGTGGG | GCAGGTTCTTGGTCACTGTGAG |
| p16-Ink4a TSS | GATGGAGCCCGGACTACAGAAG | CTGTTTCAACGCCCAGCTCTC |
| p16 exon2/3 | TTCCCAGGAGCTGAAATTCCAG | AAAAATTCCCAACACCCACTTG |
| Albumin ENH | ACCTGCGTTACAGCATCCAC | TGCTGACAGAGCAGGAGACA |

**Supplemental Table S1.** **List of qPCR primers used for the detection of histone modifications and PolII at specific genomic loci in miniChIP assays.**

**References**

1. Bolstad BM, Irizarry RA, Astrand M, Speed TP. A comparison of normalization methods for high density oligonucleotide array data based on variance and bias. Bioinformatics. 2003 Jan 22;19(2):185-93.

2. Peng S, Alekseyenko AA, Larschan E, Kuroda MI, Park PJ. Normalization and experimental design for ChIP-chip data. BMC Bioinformatics. 2007;8:219.
